# Salivary and Nasal Detection of the SARS-CoV-2 Virus After Antiviral Mouthrinses (BBCovid): A structured summary of a study protocol for a randomised controlled trial

**DOI:** 10.1186/s13063-020-04846-6

**Published:** 2020-11-02

**Authors:** Florence Carrouel, Stéphane Viennot, Martine Valette, Jean-Marie Cohen, Claude Dussart, Denis Bourgeois

**Affiliations:** 1 University of Lyon, University Claude Bernard Lyon 1, Laboratory “Systemic Health Care”, EA4129, 69008 Lyon, France; 2 National Reference Center for Respiratory Viruses, Department of Virology, Infective Agents Institute, North Hospital Network, Lyon, France; 3 Open Rome (Organize and Promote Epidemiological Network), Paris, France

**Keywords:** COVID-19, Randomised controlled trial, protocol, mouthwash, β-cyclodextrin, citrox®, salivary and real-time PCR

## Abstract

**Objectives:**

- To describe the evolution of the SARS-CoV-2 salivary viral load of patients infected with Covid-19, performing 7 days of tri-daily mouthwashes with and without antivirals.

- To compare the evolution of the SARS-CoV-2 nasal and salivary viral load according to the presence or absence of antivirals in the mouthwash.

**Trial design:**

This is a multi-center, randomised controlled trial (RCT) with two parallel arms (1:1 ratio).

**Participants:**

Inclusion criteria

- Age: 18-85 years old

- Clinical diagnosis of Covid-19 infection

- Clinical signs have been present for less than 8 days

- Virological confirmation

- Understanding and acceptance of the trial

- Written agreement to participate in the trial

Exclusion criteria

- Pregnancy, breastfeeding, inability to comply with protocol, lack of written agreement - Patients using mouthwash on a regular basis (more than once a week)

- Patient at risk of infectious endocarditis

- Patients unable to answer questions

- Uncooperative patient

The clinical trial is being conducted with the collaboration of three French hospital centers: Hospital Center Emile Roux (Le Puy en Velay, France), Clinic of the Protestant Infirmary (Lyon, France) and Intercommunal Hospital Center (Mont de Marsan, France).

**Intervention and comparator:**

Eligible participants will be allocated to one of the two study groups.

Intervention group: patients perform a tri-daily mouthwash with mouthwash containing antivirals (β-cyclodextrin and Citrox®) for a period of 7 days. Control group: patients perform a tri-daily mouthwash with a placebo mouthwash for a period of 7 days.

**Main outcomes:**

Primary Outcome Measures: Change from Baseline amount of SARS-CoV-2 in salivary samples at 4 and 9 hours, 1, 2, 3, 4, 5 and 6 days. Real-time PCR assays are performed to assess salivary SARS-CoV 2 viral load.

Secondary Outcome Measures: Change from Baseline amount of SARS-CoV-2 virus in nasal samples at 6 days. Real-time PCR assays are performed to assess nasal SARS-CoV-2 viral load.

**Randomisation:**

Participants meeting all eligibility requirements are allocated to one of the two study arms (mouthwash with β-cyclodextrin and Citrox® or mouthwash without β-cyclodextrin and Citrox®) in a 1:1 ratio using simple randomisation with computer generated random numbers.

**Blinding (masking):**

Participants, doctors and nurses caring for participants, laboratory technicians and investigators assessing the outcomes will be blinded to group assignment.

**Numbers to be randomised (sample size):**

Both the intervention and control groups will be composed of 103 participants, so the study will include a total of 206 participants.

**Trial Status:**

The current protocol version is 6, August 4^th^, 2020. Recruitment began on April 6, 2020 and is anticipated to be complete by April 5, 2021.

As of October 2, 2020, forty-two participants have been included.

**Trial registration:**

This trial was registered on 20 April 2020 at www.clinicaltrials.gov with the number NCT04352959.

**Full protocol:**

The full protocol is attached as an additional file, accessible from the Trials website (Additional file 1). In the interest in expediting dissemination of this material, the familiar formatting has been eliminated; this Letter serves as a summary of the key elements of the full protocol.” The study protocol has been reported in accordance with the Standard Protocol Items: Recommendations for Clinical Interventional Trials (SPIRIT) guidelines (Additional file 2).”

**Supplementary Information:**

The online version contains supplementary material available at 10.1186/s13063-020-04846-6.

## Supplementary Information

**Additional file 1.** Full Study Protocol.

**Additional file 2.** SPIRIT 2013 Checklist: Recommended items to address in a clinical trial protocol and related documents.

## Data Availability

Individual patient data will be kept confidential and no-account personal identifiers of the study participants disclosed to the public. Only principal study coordinator and clinical trial unit or its auditing team will have the access to the study data. To ensure confidentiality, data dispersed to project team members will be blinded of any identifying participant information.

