# Supplementary material for: Salivary and Nasal Detection of the SARS-CoV-2 Virus After Antiviral Mouthrinses (BBCovid): A structured summary of a study protocol for a randomised controlled trial
Source: Trials. 2020 Nov 2;21:906. doi: 10.1186/s13063-020-04846-6 (PMC7604647; doi:10.1186/s13063-020-04846-6)
Supplement: Supplementary file 1 — Additional file 1. Full Study Protocol. [file 13063_2020_4846_MOESM1_ESM.pdf]

Salivary and nasal detection of the SARS-CoV-2 virus after antiviral mouthrinses (BBCovid)[(1)]

Study protocol registered at www.clinicaltrials.gov with the number NCT04352959[(2a and 2b)]

Protocol version 6.0 dated 4.08.2020[(3)]

CARROUEL Florence, PhD
Associate Professor, Research
Laboratory "Systemic Health Care", EA4129
University of Lyon
florence.carrouel@univ-lyon1.fr

VIENNOT Stéphane, PhD
Associate Professor, Research
Laboratory "Systemic Health Care", EA4129
University of Lyon
stephane.viennot@univ-lyon1.fr

VALETTE Martine, PhD
Research
National Reference Center for Respiratory Viruses
Civil Hospices of Lyon
martine.valette@chu-lyon.fr

COHEN Jean-Marie, M.D
Research
Open Rome - Organize and Promote Epidemiological Network-
jmcoco@openrome.org

DUSSART Claude, PhD
Research
Laboratory "Systemic Health Care", EA4129
University of Lyon
claude.dussart@univ-lyon1.fr

BOURGEOIS Denis, PhD
Professor, Research
Laboratory "Systemic Health Care", EA4129
University of Lyon
denis.bourgeois@univ-lyon1.fr

Funding[(4)]: This work was supported by Curaden AG, Switzerland

Roles and responsibilities[5a]:

Investigators	Roles and responsibilities
**CARROUEL Florence**	Concept, protocol design, manuscript writing, critical review and overall supervision of the study. Responsible for reporting protocol amendments.
**VIENNOT Stephane**	Literature review, manuscript writing, coordination with CTU and supervision of data collection.
**VALETTE Martine**	Data collection management and samples PCR analysis and results interpretation.
**COHEN Jean-Marie**	Protocol writing
**DUSSART Claude**	Manuscript review and writing
**BOURGEOIS Denis**	Study conception and execution,

Trial sponsor[5b]: EZUS, University Lyon1, France

Role of study sponsor and funders[5c]: The sponsor and funder have no role in the study design, collection, analysis and interpretation. Curaden AG, Switzerland funded the production of mouthwash samples and contributed to the financing of the logistic hospital expenses for the study through the sponsor.

Trial coordination center[5d]: Laboratory "Systemic Health Care", EA4129, Faculty of medicine Laennec, University of Lyon, France.

Salivary and nasal detection of the SARS-CoV-2 virus after antiviral mouthrinses (BBCovid)

INTRODUCTION

Background and rational[6a and 6b]

Given the lack of effective treatment for COVID19 patients, it is necessary to explore alternative methods to contain the spread of infection, focusing in particular on its mode of transmission.

The modes of person-to-person transmission of SARS-CoV-2 are direct transmission, such as sneezing, coughing, transmission through inhalation of small droplets, and contact transmission, such as contact with nasal, oral and ocular mucous membranes. SARS-CoV-2 can also be transmitted directly or indirectly through saliva, and the fetal-oral route can also be a possible person-to-person transmission route (Jayaweera et al. 2020). In addition, high viral loads have been found in the oropharynx of infected patients, as well as in asymptomatic subjects. This may suggest that the potential for transmission of SARS-CoV-2 is broader. The oral cavity is therefore directly associated with the evolutionary process of SARS-CoV-2 in its inhalation of ambient airborne particles and its sputum.

While PCR analysis is reliable to detect the presence of viruses, swabs taken with a large cotton swab may fail to recover the virus present in the oropharynx. Saliva is a non-invasive specimen for diagnosis, surveillance and infection control in COVID19 patients. In the only published preliminary study on this subject, the SARS-CoV-2 was detected in saliva collected from 91.7% (11/12) of patients. Serial saliva viral load monitoring generally showed a decreasing trend. Live virus was detected in saliva by virus culture. The median viral load of the first available saliva samples was 3.3×10^6 copies/mL (range, 9.9×10^2 to 1.2×10^8 copies/mL)(To et al,. 2020).

The realization of cosmetic mouthwashes with or without antiviral action are "adjuvant" treatments that are part of the usual care. Therapeutic mouthwashes contain active ingredients designed to modify the dysbiotic oral microbiota. A new generation of therapeutic mouthwashes developed to control virulent bacteria has added metals, metal oxides and other nanoparticles, which appear to be promising alternatives due to their distinct physico-chemical properties.

Flavonoids are an important category of natural products and include various subgroups such as flavones, chalcones, isoflavones and flavonols. Bioflavonoids are phenolic hydroxylated structures that have been synthesized from plants and have been shown to be active against fungi, bacteria and viruses. Flavonoids as inhibitors of the chymotrypsin-like coronaviral protease have an essential function for coronaviral replication and also have an additional function for the inhibition of innate immune responses of the host and should be useful in the control of COVID-19 (Jo et al., 2019). In addition, as SARS-CoV-2 is vulnerable to oxidation, it may be of interest to use a mouthwash containing oxidizing agents such as Citrox (flavanoids) to reduce the salivary viral load of oral microbiotes, including the potential carriage of SARS-CoV-2.

Amphiphilic nanoparticles of β-cyclodextrin (C42H70O35) have been added to the composition of commercial mouthwashes to prevent the influx of hyaluronic acid in the combination of Chlorhexidine-Polylysine, Hyaluronic Acid. Amphiphilic CDs, useful for solubilizing, stabilizing or releasing intermediate sized molecules, were produced synthetically to solve the multiple difficulties of parent cyclodextrins that limit their pharmaceutical uses.

Amphiphilic CDs appear to be indicated to reduce the salivary load of oral microbes. Similarly, these modified sugar molecules would attract viruses before irreversibly inactivating them. By disrupting the outer envelope of a virus, they can destroy infectious particles by simple contact, rather than simply blocking viral growth (Jones et al., 2020). This property of β-CDs can potentially be exploited for the reduction of the viral load in the oral cavity with the use of disinfectant solutions.

Thus, the use of a mouthwash containing cyclodextrins combined with Citrox could be a valuable adjunct treatment in the fight against the COVID19 pandemic (Carrouel et al., 2020). Mouthwashes containing these two elements are marketed in Europe and could reduce salivary and nasopharyngeal viral load.

Carrouel, F. Conte, M.P.; Fisher, J.; Gonçalves, L.S.; Dussart, C.; Llodra, J.C.; Bourgeois, D. COVID-19: A Recommendation to Examine the Effect of Mouthrinses with β-Cyclodextrin Combined with Citrox in Preventing Infection and Progression. J. Clin. Med. 2020, 9, 1126.

Jayaweera M, Perera H, Gunawardana B, Manatunge J. 2020. Transmission of COVID-19 virus by droplets and aerosols: A critical review on the unresolved dichotomy. Environ Res. 188:109819. Jo, S.; Kim, H.; Kim, S.; Shin, D.H.; Kim, M.-S. Characteristics of flavonoids as potent MERS-CoV 3C-like protease inhibitors. Chem. Biol. Drug Des. 2019, 94, 2023–2030.

Jones, S.T.; Cagno, V.; Janeček, M.; Ortiz, D.; et al. Modified cyclodextrins as broad-spectrum antivirals. Sci. Adv. 2020, 6, eaax9318.

To, K.K.-W.; Tsang, O.T.-Y.; Chik-Yan Yip, C et al,. et al. Consistent detection of 2019 novel coronavirus in saliva. Clin. Infect. Dis. 2020, ciaa149.

Objectives[(7)]

Primary objective

To describe the evolution of the SARS-CoV-2 salivary viral load of patients infected with Covid-19, performing 7 days of tri-daily mouthwashes with and without antivirals.

Secondary objective

To compare the evolution of the SARS-CoV-2 nasal and salivary viral load according to the presence or absence of antivirals in the mouthwash.

Trial design[(8)]

This is a multi-center, randomized controlled trial (RCT) with two parallel arms (1:1 ratio).

METHODS: PARTICIPANTS, INTERVENTIONS, AND OUTCOMES

Study setting[(9)]

This study is multi-centered and will be carried out in 3 hospital centers, France. Each center will recruit patients for both the active and control groups. The following centers will be participating in this study:

1. Hospital Center Emile Roux, Le Puy en Velay
2. Clinic of the Protestant Infirmary, Lyon
3. Intercommunal Hospital Center, Mont de Marsan

Eligibility criteria[10]

Inclusion criteria

- Age between18 years and 85 years old.
- Clinical diagnosis of COVID-19 infection by the patient's general practitioner or hospital physician.
- Beginning of clinical signs less than 8 days ago.
- Virological confirmation of Covid-19.
- Understanding and acceptance of the trial.
- Written agreement to participate in the trial.

Exclusion criteria

- Pregnant or breastfeeding patients
- Inability to comply with the protocol
- Use of mouthwash regularly (more than once a week)
- Inability to answer questions – uncooperative
- No written agreement

Interventions

Groups[11a]

- Group test: Participants perform 3 daily mouthwashes (morning, noon and evening) with mouthwash containing β-cyclodextrin and Citrox® for 30 seconds during 7 days
- Group control: Participants perform 3 daily mouthwashes (morning, noon and evening) with mouthwash without β-cyclodextrin and Citrox® for 30 seconds during 7 days

Discontinuation of intervention[11b]

With respect to safety and selection of study participants, every effort should be made to ensure that the participant completes the clinical investigation.

Participants may be excluded from the clinical investigation and evaluation at any time. Specific reasons may justify the exclusion of a patient:

- Voluntary interruption by the participant who is free to terminate participation in the study at any time, without prejudice to the continuation of treatment.
- Constrained interruption when the state of health of participant no longer allows the protocol to be continued.
- Serious violations of the clinical investigation plan identified by the investigator and/or sponsor. These may include, but are not limited to, failure to rinse with the solution as directed, etc.
- Unauthorized admission, i.e., the participant does not meet the inclusion/exclusion criteria required for the study.
- Any adverse event or disease that, in the opinion of the investigator, may prevent continued participation in the study.
- Study-specific withdrawal criteria listed below: allergic reaction or irritation to any of the solutions, development of oral inflammatory disease, pregnancy

Clinical data collected prior to cessation or prior to the appearance of a withdrawal criterion should be analyzed.

Compliance on intervention[11c]

A diary will be completed daily by participants to verify adherence to the protocol.

Concomitant care[11d]

Study participants will continue to receive the routine medical/pulmonological care they require, according to individual requirements, under the care of their physician(s).

Outcomes[12]

Primary outcome measures

Change from Baseline amount of SARS-CoV-2 in salivary samples at 4 and 9 hours, 1, 2, 3, 4, 5 and 6 days. Real-time PCR assays are performed to assess salivary SARS-CoV-2 viral load.

Secondary outcome measures

Change from Baseline amount of SARS-CoV-2 virus in nasal samples at 6 days. Real-time PCR assays are performed to assess nasal SARS-CoV-2 viral load.

Participant timeline[13]

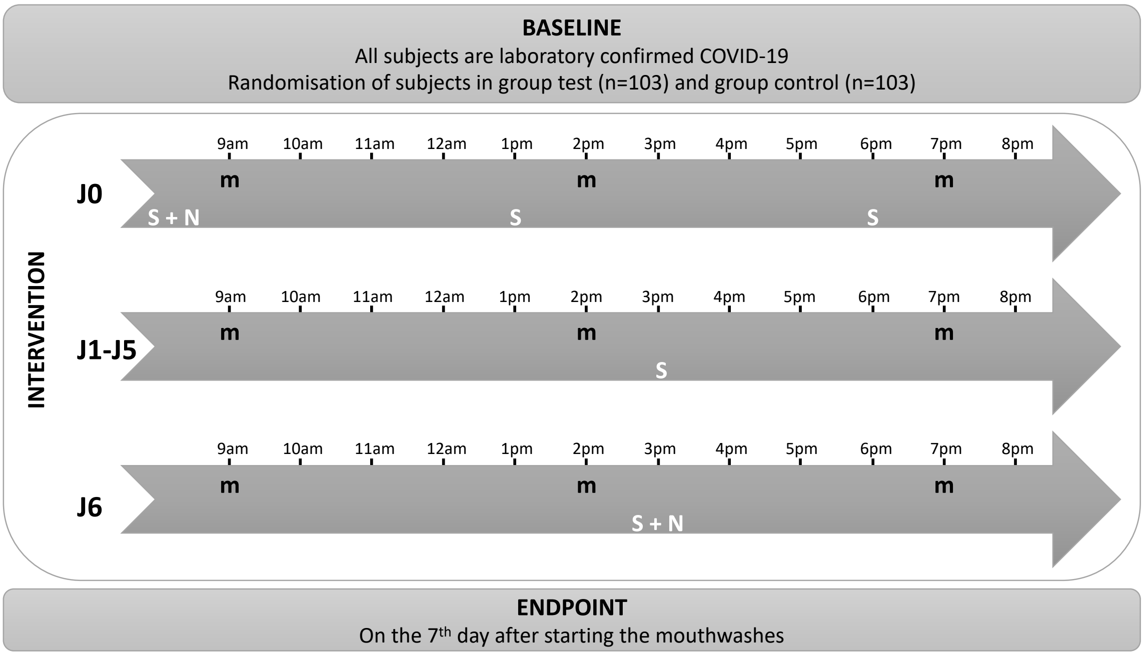

Figure 1. Flow diagram of study-participants' timeline

The numbers represent the hours of the day. m: participants realize the mouthwash. N: quantification of the SARS-CoV-2 nasal load by real-time PCR. S: quantification of the SARS-CoV-2 salivary load by real-time PCR.

Sample size[14]

The reduction in SARS-CoV-2 salivary was considered the primary outcome variable, and an estimate of a mean difference in reduction was used to calculate the sample size when two-sided differences in means between the test and control (treatment effect) groups were 5%, with a standard deviation of 10%. Using this estimate with an alpha risk of 5% and a statistical power of 90% resulted in a required number of participants per group of 86. Assuming a potential drop-out rate of 20%, 103 participants per group were determined as the target for inclusion.

Recruitment[15]

Participants are recruited through the 3 hospital centers among patients presenting for COVID-19 PCR screening and hospitalized patients with a validated COVID-19 diagnosis. If participants agree to provide their contact information to the medical investigators, they are informed of the research by phone or, verbally in case of hospitalization and the inclusion criteria are checked.
All selected adults receive in an interval of 1 day after the positive PCR results, a phone call invitation with a summary of the study objectives. Trained interviewers briefly introduce and describe the study and determine eligibility (assessment of the inclusion/exclusion criteria). Investigators inform participants that they are expected to take 3 mouthwashes per day for 7 days, to collect a saliva sample 3 times on the first day and once on the last 6 days at 3pm. On the 1st and 7th day, in addition to these saliva samples collected by the participants, a nasopharyngeal swab is performed by qualified nurses. Written consent is obtained and participants are informed that they may leave the study at any time, without this affecting their invitation to the program.

METHODS: ASSIGNMENT OF INTERVENTIONS

Allocation[16a]

Sequence generation[16a]

Participants are randomly assigned to either control or experimental group with a 1:1 allocation as per a computer generated randomisation schedule stratified by site.

Allocation concealment mechanism[16b]

Randomization is performed by the trial administrator. All participants are randomized by using the Voozalyon Didactitiel Clinical software, an on-line central randomization system. The patient number is automatically generated by software. The random allocation sequence is generated via the e-CRF with no contact with the participants. The investigator responsible for the recruitment of participants is not aware of the participant's allocation.
The trial administrator is in charge of setting up and testing the randomization process, incorporating study center stratification using 1:1 randomized blocks, ensuring that study categories of approximately the equivalent size are produced.

Implementation[16c]

After checking for all inclusion and non-inclusion criteria by the study investigator, participants are randomized to either of the study arms (intervention or control). Allocation concealment is guaranteed as the randomization is not released until the participant are recruited into the trial, taking place after all baseline measurements have been done.

Blinding[17a and 17b]

Participants, doctors, nurses caring for participants and investigators assessing the outcomes are blinded to group assignment. The data analysts are blinded to intervention status.

Unblinding

Unblinding should occur only in exceptional circumstances in case of any adverse events (AE) which will be reported immediately to the principal investigator

METHODS: DATA COLLECTION, MANAGEMENT, AND ANALYSIS

Data collection methods(18a and 18b)

Data are collected or reported in an electronic case report form (e-CRF) developed using the Voozalyon Didactitiel Clinical software to control for data quality at entry. The connection is via a username and password unique to each specific user and giving access only to the data of the centers' user. An audit trail function is included allowing a supervision and traceability of all actions from all users.

Concerning biological analysis, we have access to specialized platforms for virological and immunologic dosing, localized in Lyon allowing great communication with the study coordinator based in Lyon.

Once a participant has been enrolled and randomized into their study category, the study site makes every effort to follow them throughout the study. In order to improve compliance and prevent loss of follow-up, we organize telephone calls that are sent out prior to the intervention to remind patients of their appointments. In addition, each assessment is scheduled at the time of patient randomization, with a patient notebook given to the patient. All patients are called 24 hours prior to their assessment to remind them of their last appointment.

After acceptance, the baseline nasopharyngeal swab and the first saliva sample are obtained on day J0 just before starting the use of mouthwashes. From then on and for 7 days, participants take 3 mouthwashes at 9am, 1pm and 7pm. At J0, 4 hours after the first mouthwash, participants collecte a saliva sample and do the same after 8 hours. Then during the following 6 days, participants perform a saliva sample at 3pm. On the 7th day, in addition to this saliva sample realized by the participants, a nasopharyngeal swab is realized by qualified nurse.
To retain maximum number of patients in the study, the data collection officer keeps the contact number of all the recruited patients, so that even if they are discharged from the hospital, they are encouraged to continue with the study protocol and present to us on the day 7 at the dedicated COVID-19 testing facility for the collection of their endpoint pharyngeal swabs.

Data management[19]

All the data are entered and recorded with the electronic case report form (e-CRF)
The patients are identified by the patient code:

- Number of the center (2 digits)
- Number of the patient in the center, by chronological order of selection (3 digits)

Source documents are original documents and participant records from which participant data are reported in the e-CRF. The investigator must commit to allow direct access to data sources in the study during inspections or audits.
The source documents that may be used to complete the e-CRF are the participant records. The investigator for each study center commits to allow direct access to the participant records during inspections or audits. The encrypted data is transmitted to the data-management center via a secure internet connection.

The eCRF is designed to capture all relevant medical information from participants included in the project. The study methodologist will have a restricted reader-only access to all data in order to monitor the progress and the quality of the data.

The following documents related to this research are archived in accordance with Good Clinical Practice (I.C.H. version 4 of May 1, 1996 and decision of November 24, 2006) for a period of 15 years following the end of the research:

- By the investigators:
  - The Protocol and possible amendments to the Protocol
  - The observation notebooks
  - Source files of participants who have signed a consent form
  - All other documents and correspondence related to the research
  - Original signed informed consent forms from participants

All these documents are under the responsibility of the investigator during the regulatory archiving period.

- By the sponsor:
  - The Protocol and possible amendments to the Protocol
  - The original of the observation notebooks
  - All other documents and correspondence related to the research
  - A copy of the signed informed consents of the participants
  - Documents related to serious adverse events

All these documents are under the responsibility of the sponsor for the regulatory archiving period.

Statisticals methods(20a, 20b and 20c)

The statistical analysis consists of three main steps, namely the production of descriptive summaries of the data, modeling the data using a mixed (linear) model, and assessing correlations between viral abundances. Descriptive statistics (percentages, means and standard deviations) are calculated using SPSS 12.0 (SPSS Inc., Chicago, IL). Statistical tests (p-values) are calculated with SUDAAN 7.0 (Research Triangle Institute, Research Triangle Park, NC) for repeated measures (measures throughout the follow-up period).

For the main endpoint analysis, the mixed linear model to explain the evolution of viral quantification over time as a function of certain independent variables requires prior work on the log transform of viral quantifications. Then, in the mixed linear model, the estimated coefficients are tested for each independent variable, the null hypothesis being that the estimated coefficient is equal to zero, at the 0.05 threshold. The fixed effects are all the independent variables to be tested (sex, age, CSP, experimental group, etc.) and the random effect being the subject factor, on which the 9 salivary analyses are repeated.

The secondary endpoint -Presence yes/no of a viral load-, passes through a clustered multiple logistic regression model, with the subject effect as the cluster that will be considered for the repeated measurements. All data are considered statistically significant at $p < 0.05$.

METHODS: MONITORING

Data monitoring(21a and 21b)

The monitor must present the protocol and all procedures related to the study during an initiation visit performed before the first patient is included. A e-case report form of completion guidelines is provided to the investigator. The monitor is allowed to have access to all source documents needed to verify the entries on the eCRF and other protocol-related documents.

To ensure accurate, complete, and reliable data, the sponsor or its representatives do the following:

- Provide instructional material to the study sites, as appropriate
- Provide a start-up training session to instruct the investigator(s) and study coordinator(s). This session will give instruction on the protocol, the prompt and full completion of the clinical report forms, study procedures, and the transmission of data in a timely manner to the clinical database for statistical analyses
- Make periodic visits to the study site

- Be available for consultation and stay in contact with the study site personnel by mail, telephone, and/or fax
- Review and evaluate case report form data and use
- Conduct quality review of database

Individual data needed for the study analysis must:

- Be entered in the e-CRF as they are obtained, for both clinical and paraclinical data
- Be anonymized by the investigator
- Be authentified by an electronic signature of the investigator
- All be entered, and missing data must be justified

Routine monitoring visits are made by the monitors, designated by the sponsor to check compliance with the protocol, the completeness, accuracy, and consistency of the data and adherence to Good Clinical Practice (GCP).
The principal investigator must ensure that eCRFs are completed in a timely manner and must allow periodical access to eCRFs, patient records, drug logs, and all other study-related documents and materials.
The investigator agree to provide the monitor direct access to the subjects' source data, which may exist in the form of hospital records, patient files and notes, and laboratory assessment reports and results.

Harms[22]
Considering the very low risks in this study, there will be no formal Data Safety and Monitoring Board for this study.

Auditing[23]
An audit may be carried out at any time by persons mandated by the sponsor and independent of the research managers. Its purpose is to ensure the quality of the research, the validity of its results and compliance with the law and regulations in force.
The purpose of an audit is to confirm that the study is conducted as per protocol, International Conference on Harmonisation-Good Clinical Practice (ICH-GCP), and applicable regulatory requirements; that the well-being and the rights of the subjects enrolled have been protected; and that the data relevant for the evaluation of the investigational product have been recorded, processed, and reported in compliance with the planned arrangements. The investigators will permit a direct access to all study documents, drug accountability records, medical records, and source data.

ETHICS AND DISSEMINATION

Research ethics approval[24]
The study protocol was reviewed and approved by the French Ethics Committee "Comité de Protection des Personnes Sud Méditerranée III" (2020.04.11 six _20.04.06.46640). The protocol has been declared compliant with the MR-001 reference methodology reference frame at the National Commission of Informatics and Liberties, France (2217601 v 0).

Protocol amendments[25]
Any modifications to the protocol which may impact on the conduct of the study, potential benefit of the patient or may affect patient safety, including changes of study objectives, study design, patient population, sample sizes, study procedures, or significant administrative aspects will require a formal amendment to the protocol. Such amendment will be approved by the French Ethics

Committee “Comité de Protection des Personnes Sud Méditerranée III” prior to implementation and notified to the health authorities in accordance with local regulations.

Consent(26)
The study is explained in detail (objectives, process, expected benefits, risks...) to the participants in French (local language) by the local investigators. Written informed consent of each participant must be obtained. In addition to the clinical trial, this consent also involves the use of biological samples for laboratory testing and analysis.

Confidentiality(27)
In accordance with the French legislative data in force (articles L.1121-3 and R.5121-13 of the public health code), persons with direct access to source data will take all necessary precautions to ensure the confidentiality of information relating to the research, the persons involved and in particular their identity, as well as the results obtained. These persons, in the same way as the persons of the National Reference Center and the investigators themselves, are subject to professional secrecy.
During the course of the biomedical research or at its end, the data collected on suitable persons and transmitted to the sponsor by the investigators (or any other specialized person) will be made anonymous. Under no circumstances may the names or addresses of the persons concerned be made clear.
Coding methods: Patients, physicians and investigators will be identified by an anonymized serial number.

Declaration of interests(28)
DB declares having a consulting activity in health promotion for the funder. All other authors declare that they have no competing interests.

Access to data(29)
Only principal study coordinator and clinical trial unit or its auditing team will have the access to the study data. To ensure confidentiality, data dispersed to project team members will be blinded of any identifying participant information.

Ancillary and post-trial care(30)
The promoter of this research has taken out civil liability insurance with Chubb European Group SE ("Civil Liability" policy no. FRLSCA45275).

Dissemination policy(31a and 31b)
The trial protocol is registered at www.clinicaltrials.gov with the number NCT04352959. The results of the data analysis of this study will be published in peer-reviewed journals of dentistry and/ or medicine without disclosing any individual patient data.

APPENDICES

Informed consent(32)
A written information note in French is given to the patient. It describes the interests, objectives, conduct, benefits, risks and constraints of this study as well as the rights of the participants.

Informed consent to participate in the study is obtained in French for each participant. One consent form signed in duplicate by the participant and the investigator will be kept for record purposes.

Biological specimens[33]

The saliva and nasopharyngea samples are conserved at 4°C until the molecular analysis. All the samples are analyzed by real-time PCR. After the study, all the samples will be incinerated.
